# Behavioral and Neural correlates of Post-STROKE Fatigue: A randomized controlled trial protocol

**DOI:** 10.1371/journal.pone.0324591

**Published:** 2025-06-06

**Authors:** Kuan-Chun Liao, Isabelle Christian, Jill Stewart, Elaine Trudelle-Jackson, Wanyi Wang, Ty Shang, Hui-Ting Goh

**Affiliations:** 1 School of Physical Therapy, Texas Woman’s University, Dallas, Texas, United States of America; 2 Physical Therapy Program, Department of Exercise Science, University of South Carolina, Columbus, South Carolina, United States of America; 3 Center for Research Design and Analysis, Texas Woman’s University, Houston, Texas, United States of America; 4 Department of Neurology, University of Texas Southwestern Medical Center, Dallas, Texas, United States of America; PLOS: Public Library of Science, UNITED KINGDOM OF GREAT BRITAIN AND NORTHERN IRELAND

## Abstract

**Introduction:**

Post-stroke fatigue (PSF) is highly prevalent and lacks of effective management. Recent evidence suggest the use of transcranial direct current stimulation (tDCS) to reduce PSF. However, the effect was not lasting and the working mechanisms was unclear. The purpose of this study is to determine the behavioral and neurophysiological effects of five daily sessions of tDCS on PSF.

**Methods and analysis:**

This will be a double-blind randomized controlled trial targeting an enrollment of 32 participants with subacute-chronic stroke and significant fatigue (average Fatigue Severity Scale (FSS) > 4). Participants will be equally randomized to either anodal tDCS or sham tDCS groups. The anodal tDCS group will receive 20 minutes of 2-mA anodal tDCS applied to the ipsilesional primary motor cortex (M1) for five consecutive days. The sham tDCS group will receive the same protocol except there will be no active current delivered. Outcome assessments will take place at baseline (prior to randomization), immediately after the intervention, and at one-month follow-up. The primary behavioral outcome will be the FSS and the primary neurophysiological outcome will be an input-output curve of motor cortex excitability derived using transcranial magnetic stimulation. Secondary behavioral outcomes will include Fatigue Scale for Motor and Cognitive Function, Visual Analog Scale-Fatigue, Borg Rating of Perceived Exertion, and Paas Mental Effort Rating Scale. Secondary neurophysiological outcome will be the functional connectivity of the fronto-striato-thalamic network acquired using resting state functional Magnetic Resonance Imaging (MRI). Repeated measure ANOVA or ANCOVA will be conducted for all outcomes to compare the change between groups.

**Discussion:**

Little is known about effective treatments for PSF and the underlying mechanisms of PSF. tDCS is a promising tool to provide targeted intervention to reduce PSF symptoms. However, its lasting effect and working mechanism on PSF is elusive. The results of this clinical trial will offer critical information for PSF management and investigation.

**Trial registration:**

This trial was registered in February 1 2024 with ClinicalTrials.gov under the registration number NCT06088914.

## Introduction

Post-stroke fatigue (PSF), defined as intensified perceived effort during activities, is one of the most common complications after stroke with a prevalence as high as 65% [1]. In contrast to fatigue experienced by healthy individuals, PSF is chronic, disproportional to the intensity of activity, and does not always alleviate with rest [2,3]. PSF limits participation in rehabilitation and physical activities, consequently leading to less than optimal stroke recovery and reduced quality of life [4,5]. Given its high prevalence and significance, management of PSF has been identified as a top priority for stroke care and research [6–8].

To date, several pharmacological, physical, and psychological approaches have been examined for PSF management; however, few have demonstrated satisfactory effectiveness [9–12]. Similar to pathological fatigue in other populations, PSF is associated with increased perceived effort during activities [13–16] and altered neuroplasticity including reduced cortical excitability [17–19] and altered structural and functional connectivity [20–22]. Specifically, multiple studies have shown that reduced ipsilesional primary motor cortex (M1) excitability assessed using transcranial magnetic stimulation (TMS) is related to PSF [17–19,23,24]. This observation has motivated the use of non-invasive brain stimulation to modulate cortical excitability with the goal to improve PSF [25–30]. Transcranial direct-current stimulation (tDCS) is a widely used non-invasive brain stimulation technique that modulates cortical excitability. Anodal tDCS increases cortical excitability and cathodal tDCS has the opposite effect [31]. Previous tDCS studies have supported its efficacy in motor and cognitive recovery after stroke [32,33]; however, its use for PSF has only been recently explored. A study conducted by De Doncker et al. reported that a single session of anodal tDCS over ipsilesional M1 improved PSF as measured by the Fatigue Severity Scale (FSS) 1 week post-intervention [27]. However, the positive effect did not persist to the 1-month follow-up and no correlation was found between changes in brain excitability and changes in the FSS score. Other studies have investigated the effects of anodal tDCS to left dorsolateral prefrontal cortex on PSF but the results were equivocal and the mechanism unclear [25,26]. Therefore, while tDCS holds promise as a treatment for PSF, the working mechanism of its therapeutic effects is largely unknown.

Studies have suggested that PSF involves neural networks beyond M1 [20–22]. A pharmacological trial showed that functional connectivity of the fronto-striato-thalamic network predicted Modafinil induced PSF reduction [34]. Fronto-striato-thalamic network has been implicated in other pathological fatigue [35–37] and is likely to play a role in PSF [38]. Further, because the fronto-striato-thalamic network is anatomically and functionally connected to M1, altered M1 excitability after stroke is likely related to connectivity in this network [39,40]. Therefore, tDCS applied to M1, in addition to changing M1 excitability, might also modulate the fronto-striato-thalamic network and result in reduced PSF. Examining both brain excitability and connectivity might shed light on the underlying mechanism of PSF and the working mechanism of tDCS on PSF.

The purpose of this double-blind, randomized controlled trial is to investigate the effects of multiple sessions of anodal tDCS to ipsilesional M1 on PSF and underlying neurophysiology. We hypothesize that compared to the sham group, the anodal tDCS group will show a greater decrease in fatigue and the effect will last up to 1 month (Aim 1). We also hypothesize that compared to sham tDCS, anodal tDCS will significantly increase ipsilesional M1 excitability and the functional connectivity between ipsilesional M1 and the fronto-striato-thalamic network (Aim 2). Lastly, we hypothesize that individuals who show a greater change in the neurophysiological outcomes (M1 excitability, brain connectivity) will demonstrate a greater reduction in fatigue (Aim 3).

## Materials and methods

### Study design and setting

**Fig 1:**
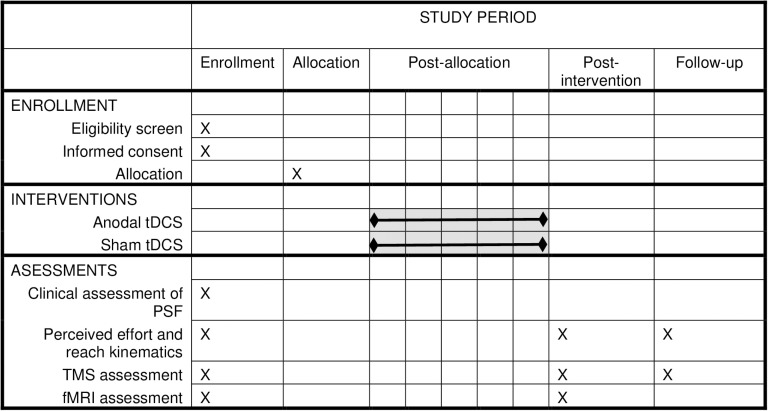
Schedule of enrollment, interventions and assessments.

**Fig 2:**
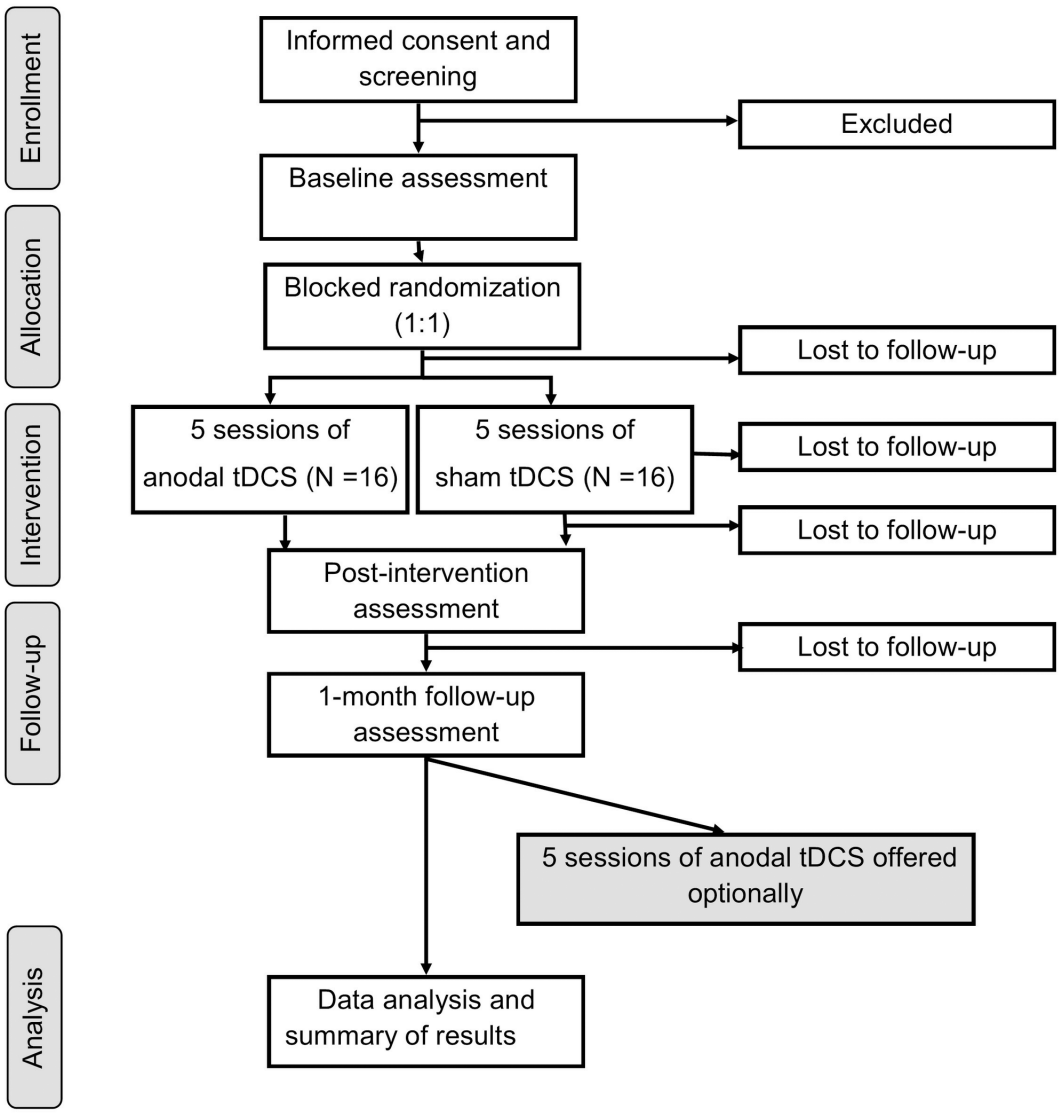
Consort flow diagram of enrollment, intervention and assessment.

The study is ongoing and we anticipate that participant recruitment will be completed in May 2026, data collection to be completed in July 2026, and resulted are expected in October 2026. This study is a two-arm double-blind randomized controlled trial. Eligible participants will be randomized to either anodal or sham tDCS groups and measurements will be taken at baseline, post-intervention, and one-month follow up. To facilitate recruitment and retention, participants will be given the option to receive five additional sessions of tDCS after the follow-up assessment in which all participants will receive anodal tDCS. The trial adheres the SPIRIT schedule (Fig 1) and will be reported using the CONSORT guidelines (Fig 2). The study will be conducted in a university-based laboratory. This trial is registered on Clinicaltrials.gov (NCT06088914).

### Participants

The inclusion criteria are: at least 18 years old; have a history of unilateral stroke ≥ 3 months prior to enrollment; have an average score ≥ 4 on the FSS [41]; have some movement capability in the more affected arm (Fugl Meyer Upper Extremity [42] ≥ 28) to ensure ability to perform the reaching task; and be able to follow three-step commands. Exclusion criteria are: acute medical problems; presence of any contraindication to tDCS, MRI or TMS; significant pain that interferes with arm movements; use of medication which may affect the level of fatigue; and presence of significant depression (Patient Health Questionnaire-9 > 10) [43].

### Sample size estimation

Previous tDCS studies on pathological fatigue reported medium-large effect sizes (f > 0.35) [25,27,57,58]. A *prior* power analysis (G*Power version 3.1.9) using a more conservative effect size (f = 0.25), a power of 0.80, α-level at 0.05, and a correlation among the repeated measures of 0.5 indicate that a minimum of 28 participants is required for repeated measures analysis of variance (ANOVA). With consideration of a 10% attrition rate, a sample size of 32 (N = 16 per group) will be required.

### Recruitment

This is a single-site study with a target enrollment of two participants per month, totaling 32 participants over 18 months. The study site is located within a medical strict in a metropolitan area. The research team has established partnership with the clinical sites within the district. Further, the study site houses a stroke center which will serve as a primary referral source. Collaborating physicians and therapists will refer patients to enroll in our study. Additional recruitment effort include posting on social media (e.g., Facebook) and printed flyers in different clinics. Financial compensation will be provided upon study completion to enhance recruitment. The start date of the recruitment period for this study was 15/12/2023.

### Randomization and blinding

Eligible participants will be randomized into either the anodal or sham tDCS group using a blocked randomization procedure. The blocked randomization will create groups that have equal numbers of females because sex is a determinant of PSF [44,45]. The study biostatistician who is not involved in intervention or assessments will assign the participants and prepare concealed envelopes with group allocation. The concealed envelopes will be given to the interventionists who will provide tDCS intervention but will not participate in the assessments. Participants and outcome assessors will be blinded to group assignment.

### Intervention

All participants will complete five daily sessions of tDCS (NeuroConn DC-Stimulator, Neurocare Group, Germany). A 0.9% saline-soaked anodal electrode (5 x 7 cm) will be placed over the ipsilesional M1 (hotspot of the affected first dorsal interosseous muscle, FDI); the cathodal electrode (5 x 7 cm) will be placed over the contralateral supraorbital area. The FDI hotspot will be determined at baseline using standard TMS protocols [46]. Participants in the anodal tDCS group will receive 2 mA stimulation for 20 minutes with a ramp-up and ramp-down time of 30 s at the beginning and end of the stimulation. The sham group will receive 2 mA electrical stimulation for 30 seconds after which the current will be ramped down to zero. This tDCS protocol complies with current safety recommendations and is in line with previous investigations examining the effects of tDCS on pathological fatigue [25,27,31,47]. Participants will remain seated during the stimulation and be instructed to minimize arm movements.

All interventionists will receive standardized training from the principal investigator (PI). A detailed intervention log will be completed at each session. The log includes information about electrode placement, start and end times of stimulation, and any deviations from the protocol. After each tDCS session, the interventionist will document the type and severity of any side effects. The PI will review all intervention logs and discuss protocol deviations and side effects with the interventionists. After the last study assessment (follow-up), participants will be asked whether they believe they were in the anodal or sham group and rate their level of confidence in their group assignment (scale from 1 to 10) [48].

All participants will be asked to adhere to their regular medications and therapies unless they will instructed otherwise by their care providers. Any changes in their care plans will be documented.

### Assessments and outcomes

All participants will take part in clinical, behavioral, and neurophysiological assessments at baseline, post-intervention, and one-month follow-up. All assessors will be blinded to group assignment and trained by the PI prior to study commencement. A detailed manual of procedure has been developed to outline all assessment procedures.

#### Clinical and behavioral assessments and outcomes.

The primary outcome for fatigue will be the Fatigue Severity Scale (FSS) score. FSS is a self-report questionnaire measuring the severity and impact of fatigue [41]. The FSS contains nine items rated on a 7-point Likert scale (1 = strongly disagree and 7 = strongly agree). The total score ranges from 9 to 63 with a higher score indicating greater fatigue. The FSS has been validated in chronic stroke [49]. Secondary measures of fatigue will be the Fatigue Scale of Motor and Cognitive Functions (FSMC) and Visual Analog Scale-Fatigue (VAS-F). The FSMC is a self-report questionnaire developed to measure cognitive and motor fatigue separately [50] and has been validated in chronic stroke [51]. The FSMC contains 20 items (10 for cognitive fatigue and 10 for motor fatigue) rated on a 5-point Likert scale. The total score ranges from 20 to 100 with a higher score indicating greater fatigue. The Visual Analog Scale-Fatigue (VAS-F) is a numerical scale ranging from 1-10 with a higher value indicating a higher level of global fatigue [52].

We previously showed that individuals with PSF reported high perceived effort during reaching and exhibited poor reach performance [16]. In the current study, we will adopt the same reaching task to evaluate changes in perceived effort and reach performance as a secondary measure of fatigue. At each assessment time point, participants will perform goal-directed reaches as fast and accurately as possible toward four targets that vary in distance and direction on a digitized tablet in sitting. Participants will also reach toward two central targets at both self-selected and fast speeds (i.e., as fast as possible) to evaluate the discrepancy between their movement choice (self-selected) and their capability (fast) [16]. Borg Rating of Perceived Exertion (Borg RPE) [53] and Paas Mental Effort Rating Scale (Paas MERS) [50] will be used to quantify perceived physical and mental effort associated with reaching, respectively. Borg RPE ranges from 6–20, with a higher value indicating greater perceived physical exertion. Paas MERS is a 9-point Likert scale that ranges from 1 (very, very low mental effort) to 9 (very, very high mental effort).

#### Neurophysiological assessments and outcomes.

A TMS device (The Magstim Company Ltd, Whitland, UK) will be used to quantify changes in cortical excitability. Surface electromyography electrodes will be placed on the affected FDI and triceps muscles while participants are seated. Each participant’s structural brain MRI will be used to guide TMS coil placement using the Brainsight Neuronagivation system (Rogue Research, Canada). For each muscle, the following TMS outcomes will be derived: resting motor threshold (RMT), motor evoke potential (MEP) amplitude, input-output curve, short-interval intracortical inhibition (SICI), and intracortical facilitation (ICF).

RMT is the minimum stimulus intensity to elicit an MEP ≥ 50 µV in amplitude in at least 3 of 5 consecutive stimulations. MEP will be elicited at rest with a stimulus intensity at 120% of RMT and at 100% maximal stimulation output. The averaged peak-to-peak MEP amplitude will be calculated to index corticospinal excitability. We will also construct an input-output curve using six stimulation intensities (100–150% of RMT) to assess corticospinal excitability and motor recruitment. Both intracortical inhibition (SICI) and facilitation (ICF) of M1 will be measured with the conditioning stimulus set at 80% RMT and the test stimulus intensity set at 120% RMT. SICI will be elicited at an inter-stimulus interval of 2 ms whereas ICF will be elicited at an inter-stimulus-interval of 15 ms. The primary measure of brain neurophysiology will be the input-output curve slope obtained from the FDI with a steeper slope indicating greater excitability [54]; all other TMS measures will serve as exploratory outcomes.

The secondary measure of brain neurophysiology will be functional connectivity of the fronto-striato-thalamic network. Participants will undergo a brain MRI on a 3T Siemens scanner with a 32-channel head coil at baseline and post-intervention. High-resolution T1- and T2-weighted anatomical scans and 10 minutes of resting state functional MRI will be acquired. Data will be preprocessed (spatial distortion correction, realignment, coregistration, normalization, smoothing, detrending, removal of lesion artifacts) using a pipeline tuned for the stroke damaged brain [55]. The mean BOLD signal between brain regions will be correlated over time resulting in a first-level connectivity matrix for each participant. Pairwise correlations between the regions of interest (M1, dorsolateral prefrontal cortex, striatum, thalamus) in the fronto-striato-thalamic network will be extracted for each participant. A higher correlation coefficient indicates greater connectivity. Functional connectivity of other networks, such as sensorimotor network [19], fronto-parietal network [38], and the default mode network [56] will be explored to further examine the neurophysiological effects of tDCS on PSF.

### Data management and data analysis

Clinical and behavioral data will be collected via paper-based questionnaires and entered into the online Research Electronic Data Capture (REDCap) hosted at the Texas Woman’s University. All paper-based files will be coded and stored in a locked cabinet with files consisting of identifiable information (e.g. informed consent) kept separate from the clinical data. Kinematic data and neurophysiological data will be in electronic format. All electronic data will be stored on password-protected university servers. All data will be coded to ensure participant confidentiality throughout the study. The statistician and the principal investigator will review the data every three months to ensure completeness and data quality. The final de-identified dataset will be deposited to NICHD DASH for public access upon the completion of publications.

All data analyses will be performed in SPSS v28 (SPSS Inc., IBM, USA) by the biostatistician and significance will be set at p < .05. The percentages and patterns of missing data will be assessed to determine if pairwise deletion will be used for analysis or if an intention-to-treat strategy needs to be applied to replace missing values [57]. Comparison of baseline variables between groups will be conducted. If a baseline difference exists and judged to be a potential confound (e.g. baseline FSS score), the variable will be included as covariate in the primary analyses. Normality and outliers will then be checked to see if parametric analysis assumptions are met.

To examine effects on clinical, behavioral, and neurophysiological outcomes, we will perform a 2 group (anodal, sham) x 3 time point (baseline, post-intervention, follow-up) repeated measure ANOVA/ANCOVA. Resting state functional MRI data will be analyzed with a 2 group x 2 time point (baseline, post-intervention) ANOVA/ANCOVA. Post-hoc Bonferroni-adjusted comparisons will be completed if a main effect or interaction is detected. To examine whether individuals who show a greater change in neurophysiological outcomes demonstrate a greater reduction in PSF, Pearson or Spearman correlational analyses will be run to examine the relationship between changes in neurophysiological measures (input-output curve, fronto-striato-thalamic network connectivity) and changes in PSF (FSS). The strength of relationships will be interpreted as follows: r < 0.25 = little or no relationship; r of 0.25 to 0.5 = fair; r of 0.5 to 0.75 = moderate; and r > 0.7 = strong relationship [58].

### Ethics and monitoring

Ethical approval has been obtained from the Texas Woman’s University Institutional Review Board (FY2023−226). The PI will obtain the informed consent and all participants will provide written informed consent. An independent Data Monitor and Safety (DMS) officer will review study progress, protocol deviations, and adverse events on a quarterly basis. Adverse events will be documented and reviewed by the investigators quarterly. Serious adverse events will be reviewed immediately and reported to the IRB and DMS officer. Protocol amendments will be reported to the DMS officer, IRB and funding agency. Interim analysis is not planned for this trial. In the events of serious adverse events, participant’s group assignment will be unblinded.

## Discussion

This study will address a long-standing problem in stroke recovery, PSF, with a novel and evidence-based intervention, tDCS, and a robust double-blind randomized controlled design. Our rich dataset composed of clinical, behavioral, neurophysiological and neuroimaging data will assist in a thorough evaluation of the effects of tDCS on PSF.

PSF is a complex, multifactorial phenomenon and likely require a multimodal approach for effective management. We propose to use neuromodulation based on the association between PSF and altered brain excitability and connectivity. Modulating brain excitability via non-invasive brain stimulation not only offers an alternative to pharmacological approaches but also provides a more targeted intervention. Previously examined strategies that have been found to be effective in reducing PSF include graded physical activity [59], cognitive-behavioral training [60], and central acting pharmacological agents [61]. These strategies share a common feature, inducing neuroplasticity. Compared to non-invasive brain stimulation, these strategies are less targeted which may contribute to variability in responses. Non-invasive brain stimulation, such as tDCS, is effective in promoting motor recovery after stroke [32] and holds promise as a treatment of non-motor symptoms such as PSF. In the current study, we choose tDCS as the non-invasive brain stimulation tool because it is safe, tolerable, and produces minor transient side effects compared to other non-invasive brain stimulation techniques such as repetitive TMS, making it a promising tool to be integrated into clinical practice.

In addition to clinical evidence, our study will generate rich neurophysiological data to advance our understanding of the neural mechanism of PSF and the working mechanism of tDCS on PSF. Our unique approach of using both TMS and functional MRI will provide a comprehensive investigation of the underlying neural mechanisms. We will use TMS to assess M1 excitability and expect that changes in M1 excitability will be associated with changes in clinical outcomes. However, tDCS applied to M1 will also alter activity in remote areas [39,40], including networks that might play a role in PSF. We therefore include functional connectivity as our secondary outcome to complement the TMS measures. Using both TMS and functional MRI will provide crucial information of the underlying mechanisms of PSF, which may assist in formulating more targeted interventions in the future. Further, our design includes both clinical self-reported measures (e.g. FSS) and behavioral outcomes (e.g. perceived effort during reaching) of PSF. These clinical and behavioral outcomes will permit us to link the neurophysiological effects to behavioral manifestations.

The current study has a few limitations. We will implement tDCS as a stand-alone treatment. Previous studies in motor rehabilitation have shown that the effect of non-invasive brain stimulation is optimized when it is combined with other interventions [62,63]. The current study will establish the efficacy of tDCS on PSF in preparation for future studies that combine tDCS with other PSF management strategies. Parameters for tDCS such as the electrode sizes were selected based on safety guidelines and previous pathological fatigue investigations [25,27,64]. These parameters might not be most effective in reducing PSF. However, optimizing tDCS parameters, in our view, should be the next step once the working mechanism of tDCS in reducing PSF is better understood. Because there is no evidence to support the connection between stroke lesion characteristics and PSF [22], we will not control for lesion. However, responsiveness to tDCS might be determined by stroke lesion characteristics [65]. Thus, it is possible that some participants will not show a response to tDCS. Our protocol involves structural and functional brain MRI and TMS assessments. These data will allow us to explore factors associated with tDCS responsiveness and provide information to guide future research. Lastly, functional MRI will not be conducted at follow-up due to insufficient resources. Therefore, the long-term effect of tDCS on the brain functional connectivity will not be examined in the current trial.

## Conclusion

This double-blind randomized controlled trial will determine the behavioral and neurophysiological effects of 5 daily sessions of tDCS on PSF. Trial results will inform the clinical treatment of PSF as well as provide important information on the mechanisms of PSF and its responsiveness to tDCS.

### Clinical trial registration

This protocol is registered on Clinicaltrials.gov (NCT06088914).

## Supporting information

S1 SPIRIT Checklist. (PDF)

S2 Protocol - for publication. (PDF)
