# Supplementary material for: Behavioral and Neural correlates of Post-STROKE Fatigue: A randomized controlled trial protocol
Source: PLoS One. 2025 Jun 6;20(6):e0324591. doi: 10.1371/journal.pone.0324591 (PMC12143528; doi:10.1371/journal.pone.0324591)
Supplement: S2 — (PDF) [file pone.0324591.s002.pdf]

Submission Type: Modification Date: 8-1-2024

IRB #: IRB-FY2023-226
Title: Behavioral and Neural Correlates of Post-Stroke Fatigue
Creation Date: 7-23-2024
Status: Review Complete
Principal Investigator: Hui-Ting Goh

IRB Modification Request

Investigator/s are required to receive approval prior to implementing any changes. Changes to the study protocol may include:

- Changes in the research team
- Addition of a data collection site
- Changes in the instrument used or data collection procedures
- Changes to the target subject population or criteria for subject inclusion / exclusion

---

*required

Please provide a summary of the modification(s) requested and a rationale for this request, if appropriate.

- Please make your changes in the appropriate sections to the left.
- Please **BOLD** the text that you have changed in each section so that the modifications can be readily located.
- Attach all necessary modified documents including updated consent forms, agency approval letters if adding a site, updated/additional testing instruments, and/or training certificates for new research team members.
- If you are making changes to the attachments, please remove the original/old attachment, and upload the new attachment with the changes **highlighted**.

Adding UT Southwestern as a performance site.
We will utilize UTSW data service for recruitment purpose.

A single IRB agreement has ben executed. We are adding UT Southwestern as a performance site in this modification.

*required

Sections Requiring Modifications

Please check all sections in which modifications are made.

- 1 - Basic Information
- ✓ 2 - Research & Review
- 3 - Study Personnel
- 4 - Study Design & Methodology
- 5 - Subject Information
- 6 - Subject Recruitment
- 7 - Study Procedures
- 8 - Informed Consent
- 9 - Study Instruments
- 10 - Risks & Benefits
- 11 - Protecting the Confidentiality of Subjects

Are there any other changes to the study that cannot be listed in the sections to the left?

For Legacy studies (studies submitted prior to the Cayuse implementation), any modifications with the exception of research personnel should be listed here.

Yes

No

Additional Documents

Attach any additional documents (not listed in the sections to the left).

Goh FY2023-226 SMART IRB UTSW Cede Letter fully signed - 7-19-24.pdf

1- Basic Information

Preparing and Completing the Application

You do not have to finish the application in one sitting; the information will be saved and you may continue at a later time. As you complete the various sections of the application new sections relevant to the type of research being conducted will appear on the left-hand side; therefore, not all numbered sections may appear. You may go to another section using the menu on the left or the arrows at the bottom of each page. When adding attachments, each attachment button will allow you to add multiple documents and most common file types can be uploaded, including .pdf, .docx, and images.

Additional help information has been added throughout the form for guidance and clarity. That additional information can be found by clicking the question mark it the top-right corner of each section. You are strongly encouraged to use this feature! Once you have answered all required sections (indicated with a red asterisk), a green check mark will appear for that section (in the menu to the left). **After all required sections are marked as complete, the option to submit the application will appear at the bottom left underneath all of the sections.**

For more information about the TWU IRB submission process, IRB tracking, and Cayuse IRB Tasks, please refer to the TWU IRB Procedures, TWU IRB website, and Cayuse help features.

*required

TWU Campus

Please select your campus. Note that if you are a student, you should select your faculty advisor's campus.

✓ Dallas

Denton

Houston

*required

Is this activity research?

✓ Yes

No

Unsure

*required

Does this research involve human subjects?

✓ Yes

No

2- Research & Review Type

*required

Type of Project

Select the type of project (Check all that apply).

Thesis

Professional paper

Dissertation

Class project

✓ Faculty research

Pilot

Other

*required

Study Review Category

Indicate the level of review for this study.

Exempt

✓ Expedited

Full

Drug, Devices, and Biologics

Will the study involve administering any of the following? Check all that apply.

Drug/Supplements

Biologics

Devices

✓ None of the above

Funding Source

Have you already received funding for this research project?

✓ Yes

List the funding agency/sponsor

Name

-

- **Name - A to Z**
- **Name - Z to A**

HHS - National Institutes of Health (NIH)

If you cannot find an agency/sponsor, please enter the sponsor name

No

*required

Study Dates

Provide an estimated start and end date for this study.

*required

Start Date

This is an estimated start date. You may NOT start your study until you receive IRB approval.
04-01-2023

*required
End Date

We will use the estimated end date you provide here as a basis for your expiration date.
08-31-2026

*required
Does this study require IRB review by more than one institution?

✓ Yes

No

Will the study utilize a reliance agreement?

Yes, TWU will rely on the other institution's IRB for the review and approval of the protocol. The other institution's IRB will serve as the IRB of Record.

Yes, the other institution will rely on the TWU IRB for the review and approval of the protocol. The TWU IRB will serve as the IRB of record.

✓ No, each institution will conduct its own IRB review.

Non-TWU IRB Information

Provide the information and documents requested below.

*required

What is the name of the other institution?

Provide the name of the Non-TWU IRB.

University of Texas Dallas (
)University of Texas Southwestern Medical Center (
)

Provide name and title of the PI on the Non-TWU IRB application (If different from TWU PI).

3- Study Personnel Information

*required

What is the Principal Investigator's status at TWU?

✓ Faculty

Student

Staff

Other

Study Personnel

Note: If you cannot find a person in the people finder, please contact the IRB Office.

*required

Principal Investigator

Provide the name of the Principal Investigator of this study.

Name: Hui-Ting Goh
Organization: Physical Therapy - Dallas
Address:
Phone:
Email:

*required

Primary Contact

Provide the name of the Primary Contact of this study.

Name: Hui-Ting Goh
Organization: Physical Therapy - Dallas
Address: T
Phone:
Email:

Co-Principal Investigator(s)

Provide the name(s) of Investigator(s) for this study.

Other TWU Research Team Members

Provide the name(s) of other TWU research team members for this study. Note: If you cannot find a person in the people finder, please contact the IRB Office.

Name: Elaine Trudelle-Jackson
Organization: Physical Therapy - Dallas
Address:
Phone:
Email:

Name: Merri Johnson
Organization: Physical Therapy - Dallas
Address:
Phone:
Email:

Name: Wanyi Wang
Organization: Research and Sponsored Programs
Address:
Phone:
Email:

Name: Priya Karakkattil
Organization: Physical Therapy - Dallas
Address:
Phone:
Email:

Name: Kuan Chun Liao
Organization: Physical Therapy - Dallas
Address:
Phone:
Email:

Name: Priya Thomas
Organization: Physical Therapy - Dallas
Address:
Phone:
Email:

Name: Kaleigh Kendall
Organization: Physical Therapy - Dallas
Address:
Phone:
Email:

Name: Isabelle Christian
Organization: Physical Therapy - Dallas
Address:
Phone:
Email:

Name: Laila Pacheco
Organization: Biology
Address:
Phone:
Email:

Name: Lizbeth Tobias
Organization: Health Promotion and Kinesiology
Address:
Phone:
Email:

Name: Monica Soto
Organization: Health Promotion and Kinesiology
Address:
Phone:
Email:

Name: Avery Foreman
Organization: Health Promotion and Kinesiology
Address:
Phone:
Email:

Human Subjects Training Certificates

If a research team member has not completed the CITI human subjects training but has a current NIH certificate (must be less than 3 years old), please attach it here.

citiCompletionCertificate_Jackson.pdf

CITI_CompletionReport9712562_Merri Leigh Johnson_2021_2024.pdf

Wang.CITI training.050621.pdf

citiCompletionCertificate_Human Subject research -Karakkattil (1).pdf

CITI Certificate_Christian.pdf

CITI_Kendall.pdf

CITI_Thomas.pdf

citiCompletionCertificate_Biomedical Research (Kuan-Chun Liao).pdf

citi certificate_Pacheo.pdf

citiCompletionCertificate_12761269_59512075_Soto.pdf

citiCompletionCertificate-Lizbeth Tobias.pdf

citiCompletionCertificate_Avery Foreman.pdf

Other Non-TWU Research Team Members

Provide the name(s) and email address(es) of other Non-TWU research team members for this study.

Jill Stewart,
Ty Shang,

Please attach a human subjects training certificate for each non-TWU research team member listed.

Note: If the Non-TWU research team member has not completed the CITI human subjects training but has a current NIH certificate (must be less than 3 years old), please attach it here.

CITI GCP 5-16-2024_Shang.pdf

Stewart_citiCompletionCertificate_January2023.pdf

Other Research Personnel

Provide the name(s) of any other research personnel who will have access to study data (e.g., transcriber of recorded interviews, transcription agencies, phlebotomist, translator, survey analyst, etc.), but will not be included as part of the research team.

Attach signed confidentiality agreement form for each person listed.

Conflict of Interest

Do you or any research team member(s) participating in this study have a financial interest related to this research project?

Yes

✓ No

4- Study Purpose & Research Questions/Hypotheses/Objectives

*required

Is this study a clinical trial?

✓ Yes

No

*required

Type of Clinical Trial

Select the type of clinical trial below. Check all that apply.

✓ Randomized

*required

Describe how the randomization will be completed for this study.

A biostatistician who will not be involved in intervention or assessment will assign participants to either the anodal or sham tDCS group using a blocked randomization procedure. The blocked randomization will create groups that have equal number of females. After initial assessment (T0), a concealed envelope with group allocation will be given to the interventionists who will deliver the tDCS intervention but will not participate in the assessments. Participants and outcome testers will be blinded to the group assignment

Non-Randomized

Placebo

✓ Blinded

*required

Single-blind or Double-blind?

Single-blind

✓ Double-blind

Other

*required

Clinical Trial Phase(s)

Select the phase of the clinical trial. Check all that apply for this study.

Pilot Study

✓ Phase I

✓ Phase II

Phase III

Phase IV

N/A

*required

Study Purpose

Describe the purpose of the study and/or the rationale for conducting this study.

Post-stroke fatigue (PSF), defined as *intensified* perceived effort during activities, is reportedly as prevalent as 85% among individuals with stroke. It negatively affects participation in rehabilitation and quality of life after stroke. The mechanisms of PSF are not fully understood, thereby limiting the development of targeted interventions. Recently, an exciting and promising intervention was introduced, demonstrating the potentials of anodal transcranial direct current stimulation (tDCS) to reduce PSF. Yet, the mechanism of action responsible for the effect remains unclear.

This study aims to use a multi-modal approach to explore the behavioral and neural correlates of PSF. As a first step, we will use M1 as a proxy to explore the neural mechanism of PSF. Our central hypothesis is that upregulating M1 excitability via multiple sessions of anodal tDCS will alter brain excitability and functional connectivity, and subsequently reduce PSF with a lasting effect.

*required

Research Questions/Hypotheses/Objectives

Provide the research question(s), study hypotheses and/or study objectives.

Aim 1: Investigate the behavioral effect of 5 daily sessions of anodal tDCS over the ipsilesional M1 on PSF.

We hypothesize that PSF measured by the Fatigue Severity Scale (Hypothesis 1a) and perceived effort during reaching (Hypothesis 1b) will decrease immediately after anodal tDCS but not sham tDCS and the effect will last up to 1 month (Hypothesis 1c).

Aim 2: Investigate the neurophysiological effect of 5 daily sessions of anodal tDCS over the ipsilesional M1.

We hypothesize that compared to sham, anodal tDCS will significantly increase ipsilesional M1 excitability measured by TMS (Hypothesis 2a) and functional connectivity between ipsilesional M1 and the fronto-striato-thalamic network measured by resting state functional MRI (Hypothesis 2b).

added on Sept 19 2023: Aim 3: Determine the relationship between changes in M1 excitability, brain connectivity and changes in PSF. We hypothesize that individuals who show a greater change in M1 excitability (Hypothesis 3a) and functional connectivity (Hypothesis 3b) will demonstrate a greater reduction in PSF.

5- Subject Information

*required

Subject Enrollment

Provide a description of the subjects in this study.

32 individuals post-stroke will be recruited from local community.
We will use study flyer and word of mouth as our means of recruitment.

*required

Approximate Number of Subjects to be Enrolled

Please enter the estimated total number of subjects to be enrolled in this study.

32

*required

Vulnerable Populations

Select below any population(s) that you will specifically recruit for this study. Check all that apply. If no vulnerable populations will be recruited, check "None of the Above."

Pregnant women

Fetuses

Minors

Prisoners

Individuals with Impaired Decision-Making Capacity

Other

✓ None of the Above

*required

Age (or age range)

Provide the age or age range of study subjects.

18+

*required

Provide a rationale for the inclusion/exclusion based on age.

The proposed study will not include individuals younger than 18 years old. Prevalence of stroke is very low among individuals younger than 18 years old and usually associated with atypical causes (e.g. congenital heart disease, hematological disorders, or trauma). Children with stroke also demonstrate different clinical presentations and outcomes as adults with stroke. To ensure the validity of the proposed study, we will only enroll those older than 18 years of age.

*required

Sex of Study Subjects

Select the sex of the subjects that will be enrolled in this study.

Female

Male

✓ Both

*required

Ethnicity of Subjects

Will subjects be included/excluded based on ethnicity?

Yes

✓ No

Additional/ Other Inclusion Criteria

List any other inclusion criteria to be considered for participation in the study. Provide a rationale for all inclusion criteria listed.

1) be at least 18 years old;
2) have a history of unilateral stroke ≥ 3 months prior to enrollment;
3) have an average score ≥ 4 on Fatigue Severity Scale;
4) have some movement capability in the more affected arm (Fugl Meyer Upper Extremity ≥ 28) to ensure they can perform the reaching task;
5) be able to follow three-step commands.

Additional/Other Exclusion Criteria

List any other exclusion criteria to be considered for participation. Provide a rationale for all exclusion criteria listed.

1) acute medical problems, **Added on April 5 2023: such as active infection, undergoing cancer therapy**
2) presence of any contraindication to tDCS, MRI or TMS;
3) presence of significant depression (Patient Health Questionnaire-9 > 10);
4) significant pain that interferes with arm movements;
5) use of medication which may affect the level of fatigue.

6- Subject Recruitment

*required

Subject Recruitment

*Will this study **ONLY** utilize secondary data (is this a retrospective study)?*

Yes

✓ No

*required

Eligibility Screening/Testing

Will the study utilize screening/eligibility questionnaires, tests, forms to be completed by or administered to the subject?

✓ Yes

Please explain the process for screening subjects.

Interested participants will come to the study site-TWU-Dallas for an in-person screening.
They will first read and sign the approved consent prior to the in-person screening.
The informed consent includes an explanation of the screening process and eligibility.
Participants will be screened first with Fatigue Severity Scale and Fugl Meyer Upper Extremity.
To be eligible for the study, they need to score an average > 4 on the Fatigue Severity Scale and total score > 28 on the Fugl Meyer Upper Extremity.
They will also be screened for the TMS & tDCS safety, MRI safety and patient health questionnaire-9 for depression.

Attach any screening/eligibility testing documents.

Fatigue-Severity-Scale.pdf

Fugl-Meyer Assessment_UE.pdf

PHQ9.pdf

TMS-tDCS Safety_questionnaire.pdf

BHIC_MRI SafetyScreeningForm(v2).pdf

No

*required

Recruitment Process

Describe subject recruitment process in detail.

We will recruit 32 participants from local communities.
Below describes the recruitment process
1. Study flyers (see attached) will be posted at the local communities, such as The Stroke Center-Dallas, and social media platforms upon the approval of the platforms administrators.
2. Recruiting emails (see attached) will be sent out to previous participants who have agreed to be contacted for research.
3. Interested individuals may contact the researchers directly via email or phone for the initial screening (age, stroke chronicity).
4. Individuals with stroke will then be scheduled an in-person screening session (upon participant's preference) in which the Fatigue Severity Scale and Fugl-Meyer Upper Extremity will be administered. To be eligible, participants with stroke need to score average > 4 on the Fatigue Severity Scale and > 28 on the Fugl-Meyer Upper Extremity.
The in-person screening also including TMS, tDCS and MRI safety questionnaires, and PHQ-9 questionnare.
Upon completing the screening, the researchers will schedule the first assessment session.

Recruitment Documents/Materials

Attach all study recruitment materials you will use in this section. This includes (but is not limited to) flyers, email/phone/verbal scripts, social media posts, letters, advertisements, etc.

Flyer.pdf

Email script_revised April 5 2023.docx

Email script_revised September 19 2023.docx

Flyer_revised Sept 19 2023.pdf

Flyer_revised Dec 6 2023.pdf

Flyer_revised July 2024.docx

Email script_revised July 2024.docx

*required

Will the subjects be told about the intent of the study prior to participating?

✓ Yes

No

*required

Research Procedures

Describe the research procedures in detail.

The overall goal of this prospective randomized study is to determine the behavioral and neural effects of anodal M1 transcranial direct current stimulation (tDCS) on PSF. Eligible individuals will be randomly assigned to complete 5 daily sessions of anodal (experimental) or sham (control) tDCS to the ipsilesional primary motor cortex.

Before (T0) and immediately after the intervention (T1), clinical and behavioral assessments of PSF, brain excitability assessment using transcranial magnetic stimulation (TMS), and brain functional connectivity using resting state fMRI will be conducted by blinded assessors. A 1-month follow-up behavioral and TMS assessment (T2) will also be completed to determine if the effects of tDCS are lasting.

Below table and description detail the procedures

Added on April 6 2023

Screening and Consent (visit 1)	TO assessment (visit 1-3)	Intervention (visit 4-8)	T1 asssement (immediately after the last tDCS session) (visit 9-11)	T2 (1 month after the last tDCS session) (visit 12-13)
	• Clinical assessment<br>• Behavioral assessment<br>• MRI assessment<br>• TMS assessment	5-daily sessions of tDCS	• Clinical assessment<br>• Behavioral assessment<br>• MRI assessment<br>• TMS assessment	• Clinical assessment<br>• Behavioral assessment<br>• TMS assessment

1. Screening, randomization and double blinding: After consent, the **PI (revised on April 5 2023)** will perform screening (see Section 6). The screening and consenting process will take approximately 30 minutes. A biostatistician who will not be involved in intervention or assessment will assign participants to either the anodal or sham tDCS group using a blocked randomization procedure. The blocked randomization will create groups that have equal number of females. After initial assessment (T0), a concealed envelope with group allocation will be given to the interventionists who will deliver the tDCS but will not participate in the assessments. Participants and outcome assessors will be blinded to the group assignment.

2. tDCS intervention: After group assignment and T0 assessment, participants will come to TWU-Dallas for 5 consecutive days to receive tDCS intervention. Each intervention session will take approximately 45 minutes including preparation time and checking for possible side effects after stimulation.

tDCS will be applied using a battery-driven stimulator while participants are seated and at rest. The anodal electrode (35 cm^2) will be placed over the ipsilesional primary motor cortex (hotspot of the affected first

dorsal interosseous muscle identified using TMS) and the cathodal electrode (35 cm^2) will be placed over the contralateral supraorbital area. The real anodal tDCS group will receive 2 mA, 20 minutes of stimulation with a ramp-up and ramp-down time of 30 s at the beginning and end of the stimulation. Sham stimulation will be achieved by ramping down the current intensity immediately after ramping up (30 s) while the remaining stimulation parameters stay the same. This protocol complies with current safety recommendations and is in line with previous investigations examining the effects of tDCS on PSF.
A side effect questionnaire (see Section 9) will be filled after each tDCS session to document the frequency and type of side effects.
Added on April 5 2023: Two researchers will be assigned as interventionist and both of them are trained physical therapists. Note that tDCS is an investigational device and administration of tDCS does not require certifications or credentials. Both interventionists will be trained by the PI prior to the commencement of the study.

Procedure 3, 4 and 5 will take place at T0, T1 and T2; they will be distributed over 2 visits to avoid fatigue with testing.
3. Clinical assessment of: We will administer three clinical fatigue scales (see Section 9) at each assessment time point: Fatigue Severity Scale (FSS), Fatigue Scale of Motor and Cognitive Functions (FSMC), and Visual Analog Scale-Fatigue (VAS-F). The 9-item FSS evaluates severity and impact of fatigue on activity. The 20-item FSMC evaluates the impact of fatigue on motor and cognitive function separately. The VAS-F is a numerical scale ranging from 1-10 assessing the global level of fatigue. The clinical assessment will take about 15 minutes.

4. Behavioral assessment: Participants will perform a reach task on a digitized tablet using both their less affected and affected arms. Perceived effort will be probed using Borg Rating of Perceived Exertion (Borg RPE) and Paas Mental Effort Rating Scale (Paas MERS) (see Section 9)because pathological fatigue often manifests in both domains. Participants will also reach toward two central targets at both self-selected and fast speed (i.e., as fast as possible).

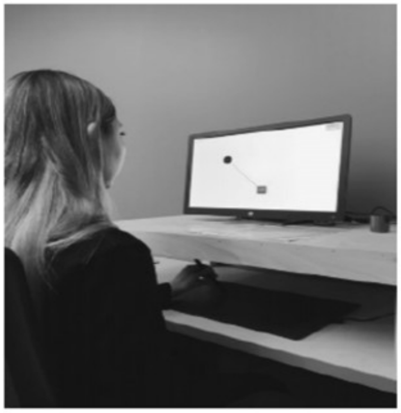

5. Assessment of brain excitability using TMS: We will use standardized single- and paired-pulse TMS protocols to assess brain excitability.
We will record motor evoked potentials from the affected first dorsal interosseous (FDI) and triceps brachii muscles. We will also construct input-output curves at 6 stimulation intensities (100-150% of resting motor

threshold, RMT). Short-interval intracortical inhibition (SICI) and intracortical facilitation (ICF) will be examined using paired pulse TMS. The inter-pulse interval will be set at 2 ms for the SICI and 15 ms for the ICF; the conditioning and test stimulus intensity will be set at 80% RMT and 120% RMT respectively. These TMS measures are selected because they are correlated with PSF based on our preliminary studies and responsive to anodal tDCS.
Added: The PI will perform the TMS assessment.

Procedure 6 will occur at T0 and T1 and will take approximately 1 hour including the preparation time.
6. Assessment of brain connectivity using resting state fMRI: Participants will go to UT Dallas BrainHealth Imaging Center for neuroimaging assessments at T0 and T1.
The center is about 1.4 mile from TWU-Dallas. A 3T Siemens scanner with a 32 channel head coil will be used to obtain high-resolution T1- and T2-weighted anatomical scans (1 mm^3 isotropic voxels). We will acquire 10 minutes of resting state fMRI (rsfMRI, TR=2000ms, FOV=220x220mm^2, 39-4 mm thick slices). The MRI scanning time will be approximately 45 minutes.
Added: The PI will coordinate the MRI scan with the UTD. The PI is trained for Level I and II MRI safety. She will screen the participants, schedule the scanning time with the imaging center, and assist with participant set-up. The relying institute, UTD, will have level III trained technician to operate the MRI scanner.

Added on Dec 7 2023:
7. Optional extended anodal tDCS (visit 14-visit 18): Participants who agree to take part in the optional extended intervention will come back to the laboratory for 5 consecutive days after they complete the T2 assessment (see above table). During these sessions, participants will receive anodal tDCS applied to the ipsilesional primary motor cortex. The anodal tDCS procedure will be identical to that outlined in procedure 2. tDCS intervention above. Similarly, we will fill out the side-effect questionnaire after each session.

8. additional assessment (visit 19-20): Those participate in the optional extended intervention will visit the laboratory for two more visits to complete the clinical, behavioral and brain excitability assessments. These assessments will be the same as outlined in procedure 3, 4 and 5.

*required

Is video recording a part of this study?

Yes

✓ No

*required

Is audio recording a part of the study?

Yes

✓ No

*required

Is internet/email a part of the study?

✓ Yes

*required

Describe how the internet and/or email will be used.

Part of the screening, e.g. checking for age and chronicity might take place via email communication.
Upon participants' request, the researcher will send a copy of study flyer or informed consent form to them so they can review prior to scheduled meetings.
Email might also be used for scheduling purpose.

No

*required

Non-TWU Study Site

Will the subjects be affiliated with a specific non-TWU agency, institution, or organization?

Yes

✓ No

*required

Location/Setting of the Study

Where will the study take place? Describe the physical and privacy aspects of this location.

The study activities will take place at the following locations/rooms
1. TWU IHS-Dallas Room 8116: This room will be used to conduct clinical and behavioral assessments. The room locates in the research suite and access is controlled; only approved personnel will have access to the room.

2. TWU IHS-Dallas Neurophysiology lab: This room is located in the Stroke Center-Dallas will be use for TMS assessment. The room's access is limited and only used for research purpose. This room will also be used for tDCS intervention. We will use a google calendar to reserve the room to avoid conflicts between assessment and intervention schedule.

3. UTD-Brain Health Imaging center: The BrainHealth Imaging Center at the University of Texas at Dallas is the secondary study site located 1.4 mile way from the IHS-Dallas. The center occupies 4300 square feet and includes a reception area and waiting room, equipment rooms, and a changing room with lockers. Two rooms are dedicated to two Siemens 3T Prisma scanners that run Syngo MR E11C software that are separated by a central control room. The center is designed for research use and use a centralized scheduling system to protect participant's privacy to the extent allowed by the regulation.

*required

Time Commitment

What is the time commitment for the subjects? Include the number of sessions/visits, maximum time commitment per session, and the maximum total time commitment.

At T0 and T1, participants will complete 3 visits of assessment (1 for MRI, 2 for behavioral and TMS). Each session lasts 1 or 2hours.
At T2, participants will complete 2 visits of assessment (1 for behavioral and 1 for TMS). Each session lasts 2hours.
Participants will also attend 5 daily intervention sessions. Each session lasts 1 hour

The total number of visits will be 13 visits.
The total time will be 19-21 hours (T0 = 5 hours; T1 = 5 hours; T2 = 4 hours; Intervention = 5 hours) including delays, breaks etc.

Added Dec 7 2023:

Participants opt in for the extended intervention will have additional 7 visits (a total of 20 visits). Their total time will be 21 hours + 9 hours = 30 hours.

*required

Subject Data, Specimens, and Records

Does this project involve the collection or use of materials (data or specimens) recorded in a manner that could identify the individuals who provided the materials, either directly or through identifiers linked to these individuals?

✓ Yes

No

8- Informed Consent Process

*required

Questions about the Study

Subjects should be provided the opportunity to ask the researchers questions about the study at any time before, during and after the completion of the study. Describe how subjects can contact you if they have questions about the study.

Interested participants will contact the PI via email or phone, they will be encouraged to ask questions related to the study during the initial process. They then go through the consent and screening process (see Section 6& 7). The researchers will remind participants their right to ask questions and request for clarification during the consent and screening process.
Throughout the study course (assessment, treatment, and follow up), participants will be reminded that they can ask questions at anytime.
After the completion of the study (after the last follow up session), participants will be provided the PI's contact and be encouraged to reach out if they have any questions.

Added on Dec 7 2023
During the consent process, participants will be asked if they would like to take part in the optional extended intervention. Those who opt in for the extended intervention will initial on the consent form (attached Consent revised Dec 2023).

*required

Does this study use signed informed consent?

This includes obtaining a signature (including electronic) on a consent form

✓ Yes

*required

Signed Informed Consent

Describe in detail the process for obtaining written informed consent.

After the initial screening (age, stroke duration) over phone, participants will come to the study site for in-person screening.
Prior to screening, participants will be given an approved written consent form.
The researcher will ask the participant if he/she prefers to read the written consent by himself/herself, or have the researcher read it to them.

Participants are encouraged to ask questions when reading the consent.
After reading the consent, researcher will ask a few questions to make sure participants fully understand the procedure, benefits and risks. These questions include but not limited to "How many time do you need to come to TWU for the study?" , "What might be a possible side effect when you are getting the tDCS treatment?"
Researcher will then remind participants that their participation is voluntary and they have the rights to withdraw at anytime.
Participants will then sign and date the written consent.
Only participants who meet the criteria will be enrolled and proceed with the other study procedures outlined in Section 7.

*required

Signed Consent Form Storage

Describe where you will securely store signed consent forms (must be in a secure location). Explain how long the consent forms will be kept (must be maintained for a minimum of three years from the study close date). Describe how you will destroy the signed consent forms after this period. Note that copies of signed consent forms must be submitted to the IRB when you submit your study close request.

All signed consent will be stored at the PI's office (IHS-Dallas Room 8621) in a locked cabinets.
The consent will be kept for 5 years from the study close date.
After that, the signed consent form will be shredded.
Electronic copies (pdf) of singed consent will be submitted to the IRB when study close request is submitted.

*required

Consent Form(s)

Attach the study consent form(s).

Goh_Behavioral and Neural Correlates of PSF_Informed Consent_revised April 6 2023.docx

Goh_Behavioral and Neural Correlates of PSF_Informed Consent_revised September 19 2023.docx

Goh_Behavioral and Neural Correlates of PSF_Informed Consent_revised December 2023.docx

Goh_Behavioral and Neural Correlates of PSF_Informed Consent_revised Jan 2024.docx

Goh_Behavioral and Neural Correlates of PSF_Informed Consent_revised July 2024.docx

No

9- Study Instruments

*required

Study Instruments

Will any data collection instruments (e.g., data collection forms, surveys, questionnaires, interviews, focus group discussion, etc.) be used in the study?

✓ Yes

*required

Please attach all data collection instruments here.

Demographic sheet.pdf

Reaching Recording Sheet.pdf

Fatigue scale for motor and cognitive functions.pdf

Fatigue-Severity-Scale.pdf

TMS assessement form.pdf

Exit questionnaire.pdf

Borg Rate of Perceived Exertion.pdf

Rating Scale Mental Effort_PAAS.pdf

tDCS side effect form.pdf

No

*required

Will these instruments record any information that can identify the subjects?

✓ Yes

*required

Please justify why the instruments need to record identifiable information.

The demographic form will contain participant's name, DOB, emergency contact along with their medical history.
The demographic form will be stored together with the screening form and signed consent as they all contain identifiable information.
The form will be kept in a locked cabinet in the PI's office separated from the other data sheets.

No

10- Risks & Benefits

*required

Potential Risks and the Steps to Minimize the Risks

*required

List all the potential risks to the human subjects involved in this research. All risks must be identified and listed on the consent form (if applicable).

The risk involved in the proposed study is minimal and entirely non-invasive.
Potentials risks include: loss of confidentiality, loss of time, fatigue, mild discomfort associated with brain stimulation and MRI protocols, and incidental findings on MRI

*required

Describe how each risk will be minimized.

1. Loss of confidentiality: The investigators will attempt to maintain confidentiality to the extent that is allowed by law. Codes, rather than names, will be used in data management and analysis, as well as in the final report. All electronic data will be coded and stored in password-protected computers. The hard copy of data records will be stored in a locked file cabinet. The data recorded on papers will be stored for approximately 5 years and then will be shredded. The data recorded on disks will be stored for 5 years and then will be deleted. It is anticipated that data will be published in books and/or journals. However, names or other identifying information will not be included in any publications. All research personnel have completed education programs and passed competency tests regarding protection of human research participants in compliance with NIH standards.

2. Loss of time: We will try our best to minimize the impact the loss of time and schedule the visits at days and times that are mutually convenient for participants and the research team. This includes scheduling sessions early in the morning, evening or weekends.

3. Fatigue: Assessment at each time point will be distributed over 2-3 days to minimize fatigue. Each visit will include scheduled rest breaks. The intervention is expected take less than an hour per day and does not involve physical or cognitive activities. Fatigue is expected to be minimal during the tDCS sessions. Participants will be reminded to request breaks whenever they feel tired. The researchers will also pause or stop the procedure if deemed necessary. **Participants who can not tolerate the protocols due to fatigue will be withdrawn from the study.**

4. Risks associated with brain stimulation: Participants will be carefully screened using a standardized safety questionnaire. Individuals with contraindications to TMS or tDCS will be excluded.
The study will use single pulse and paired pulse TMS. Unlike repetitive TMS, single pulse and paired pulse TMS have minimal risk in triggering seizures. The risks associated with single pulse and paired pulse TMS include temporary hearing threshold changes due to TMS noise. Earplugs

will be offered during the TMS protocol to minimize the discomfort associated with noise generated during TMS stimulation. Participants might develop headache or fatigue during or after TMS session. These are often temporary and usually resolve within 2-3 hours.

tDCS might result in skin reaction underneath the electrodes (itching, burning sensation or redness). A skin check will be performed 5 minutes after and at the end of the tDCS stimulation by the research personnel. Headaches, fatigue or dizziness might occur during or after tDCS. These symptoms are temporary.
A side effect questionnaire will be filled out after each session of tDCS. (see Section 9).
Participants who cannot tolerate the TMS or tDCS protocol will be withdrawn from the study.

5. Risks associated with MRI: There is a potential for discomfort due to lying still in the scanner for the duration of the scan (~45 minutes). Participants will be closely monitored while in the scanner for discomfort. Positioning for maximal comfort will be provided with a head support, bolster under knees, and cushion surrounding arms. Noise associated with MRI can be disturbing and participants will wear a noise cancelling headphone in the scanner.
Incidental findings on MRI (e.g. structural anomalies) are possible. If something appears abnormal, the neurologist consultant (Dr. Ty Shang) and **added on sept 19 2023: a radiologist from UTD** will be consulted. The participants will be informed and a copy of the MRI will be provided to the participant so that he or she can take it to a medical expert for further review and assessment. **added on sept 19 2023: No diagnosis will be made by the research team based on the incidental findings.** During the informed consent process, the possibility of incidental finding will be reviewed and discussed. If the participant does not wish to be informed of any incidental findings from the MRI, he or she should not participate in the study.

*required

Benefits/Remuneration

What will the subject receive for participating in the study?

(i.e., financial remuneration, free services, access to information, and access to an intervention) If there are none, state below that there are no direct benefits to the subject.

Participants will receive **revised on sept 19 23: $500** upon completion of the last assessment.
They also will have access to their individual assessment information.
Participants can request to receive a copy of result summary when the study is completed.

Added on Dec 7 2023:
Participants who opt in for the optional extended intervention will receive additional $200 upon completion of the last visit (visit 20).

Revised on July 15 2024:
Participants who opt in for the optional extended intervention will no receive additional incentive.

What are the generalizable benefits of this study? *(i.e., contribution to knowledge in a particular field)*

Our long-term goal is to develop evidence based, theory-driven interventions to manage post-stroke fatigue. This study investigates a relative novel intervention to mitigate post-stroke fatigue and adopts a multimodal approach to examine the underlying mechanisms. The comprehensive research will guide the development of treatment targeted the underlying mechanisms of PSF.

*required

Study Results

Will you provide results of the study to the subjects after the completion of the study?

✓ Yes

*required

Explain how *(e.g., mail, email, posting online, etc.)* you will provide the results of the study to the subjects.

Based on individual request, study results (aggerated data) will be emailed or mailed to participants when the study is completed.

No

11- Protecting the Confidentiality of Subjects

*required

Identifiable Private Information to be Collected

List all documents, recordings, electronic data, health records, biospecimens, etc., that contain identifiable private information to be collected in this study.

The safety screening forms (TMS & tDCS safety questionnaire and MRI safety form), the demographic form, and the eligibility screening form will consist of participant's name and health history.

*required

Storage Location and Protection of Identifiable Private Information

---

Where will the identifiable private information or data be stored? Describe the security measures you will take to protect the stored data.
(e.g., in a locked file cabinet with limited access, or a password protected computer.)

The safety screening forms will be stored in PI (Hui-Ting Goh) office in a locked cabinet with limited access.

Revised on May 24 2023: The MRI images will be shared with the PI on the same day of the scanning. The data will be deidentified and stored in password protected drives

*required

Electronic Transmission of Identifiable Private Information

Will the identifiable private information be transmitted electronically? (This includes, but is not limited to downloading, emailing, transferring from cloud storage to computer/hard drive/flash drive, and/or video conferencing .)

✔ Yes

*required

Explain how data will be protected during transmission.

Provide the steps/security measures you will take to protect the data during transmission.

Revised on May 24 2023: The deidentified brain MRI will be downloaded to a password protected USB drive. A backup copy of the MRI will be stored in the PI's TWU one-drive.
The MRI images stored on one drive and backup hard drive will be coded using subject ID; therefore not identifiable.

No

*required

Identifiable Private Information Destruction Timeline

Will the documents containing Identifiable Private Information be destroyed?

✓ Yes

*required

Timeline for the Destruction

Provide a time frame for when the documents containing identifiable private information will be destroyed. (e.g., 5 years after the completion of the study.)

5 years after the study closures.

*required

Method(s) of Destruction

Identify specific ways that the documents containing identifiable private information will be destroyed at the end of this period of time.

The safety screening forms will be shredded 5 years after study closures along with the signed consent forms.

No
